# Proposal for a Unitary Anatomo-Clinical and Radiological Classification of Third Mobile Window Abnormalities

**DOI:** 10.3389/fneur.2021.792545

**Published:** 2022-01-11

**Authors:** Pierre Reynard, Samar Idriss, Aicha Ltaief-Boudrigua, Pierre Bertholon, Andreea Pirvan, Eric Truy, Hung Thai-Van, Eugen C. Ionescu

**Affiliations:** ^1^Department of Audiology and Neurotology, Lyon University Hospital, Lyon, France; ^2^Department of Physiology, Claude Bernard Lyon 1 University, Lyon, France; ^3^Paris Hearing Institute, Institut Pasteur, Inserm U1120, Paris, France; ^4^Department of Otolaryngology - Head and Neck Surgery, Eye and Ear University Hospital, Beirut, Lebanon; ^5^Department of Radiology, Lyon University Hospital, Lyon, France; ^6^Department of Otorhinolaryngology, University Hospital of Saint Etienne, Saint Etienne, France; ^7^Department of Otorhinolaryngology, Lyon University Hospital, Lyon, France

**Keywords:** third window lesions, otic capsule dehiscence, classification, endovascular treatment, third window pathomechanism

## Abstract

**Introduction:** An increased number of otic capsule dehiscence (OCD) variants *relying on the third window pathomechanism* have been reported lately. Therefore, a characterization of the anatomical structures involved and an accurate radiological description of the third window (TW) interface location have become essential for improving the diagnosis and appropriate therapeutic modalities. The purpose of this article is to propose a classification based on *clinical*, anatomical, and radiological data of third mobile window abnormalities (TMWA) and to discuss the alleged *pathomechanism in lesser-known clinical variants*.

**Materials and Methods:** The imaging records of 259 patients who underwent, over the last 6 years, a high-resolution CT (HRCT) of the petrosal bone for conductive hearing loss were analyzed retrospectively. Patients with degenerative, traumatic, or chronic infectious petrosal bone pathology were excluded. *As cases with a clinical presentation similar to those of a TW syndrome have recently been described in the literature but without these being confirmed radiologically, we thought it necessary to be integrated in a separated branch of this classification as “CT - TMWA.” The same goes for certain intralabyrinthine pathologies also recently reported in the literature, which mimic to some extent the symptoms of a TW pathology. Therefore, we suggest to call them intralabyrinthine TW-like abnormalities*.

**Results:** Temporal bone HRCT and, in some cases, 3T MRI of 97 patients presenting symptomatic or pauci-symptomatic, single or multiple, unilateral or bilateral OCD were used to develop this classification. According to the topography and anatomical structures involved at the site of the interface of the TW, a *third*-type classification of OCD is proposed.

**Conclusions:** A classification reuniting all types of TMWA as the one proposed in this article would allow for a better systematization and understanding of this complex pathology and possibly paves the way for innovative therapeutic approaches. *To encompass all clinical and radiological variants of TMWA reported in the literature so far, TMWAs have been conventionally divided into two major subgroups: Extralabyrinthine (or “true” OCD with three subtypes) and Intralabyrinthine (in which an additional mobile window-like mechanism is highly suspected) or TMWA-like subtype. Along these subgroups, clinical forms of OCD with multiple localization (multiple OCD) and those that, despite the fact that they have obvious characteristics of OCD have a negative CT scan (or CT – TMWA), were also included*.

## Introduction

### History of the Third Window Hypothesis

Therapeutic fenestration is the first technique that emerged in the history of the development of the third window (TW) concept, which was implemented in the early twentieth century by the hearing loss surgeons pioneers. This technique, reserved for patients with advanced forms of otosclerosis, aimed at improving hearing status by surgically creating a new window on the lateral or superior semicircular canal (SSC) (1–5). Thus, the transmission of acoustic energy through the inner ear fluids to the cochlear receptors was partially reestablished. Although this technique did not rely on a physiological restoration of the cochlear micromechanics, many patients experienced improved hearing immediately after surgery. However, they frequently complained about the appearance of a concomitant vertigo triggered by loud sounds.

Subsequently, the animal experimental research published by Tullio proved that, in pigeons, a surgically created window on the lateral bony SC can generate endolymphatic flow leading to vestibular disorders when exposed to loud sounds (6, 7). In addition, a medium-intensity sound exposure leads to endolymphatic flow and progresses rhythmically to the crista ampullaris, generating in the experimental animal a tonic postural movement in the SC plane involved. Increasing the sound intensity would lead to a reinforced endolymphatic current, which would deform the crista in a more consequent manner. Moreover, at some point following further sound stimulation, the endolymphatic current ceases to move in an alternating rectilinear manner and begins to rotate rapidly around the longitudinal axis of the membranous SC, thus permanently deforming the crista. At that point in the experiment, Tullio noticed that the animal stopped making pendulous movements of the head, which remained fixed due to tetanic contractions of neck muscles.

As an early observation leading up to the modern period, Schuknecht reported a “mysterious” conductive deafness associated with a particular form of otosclerosis with foci located on the inner part of the round window (8). In a different setting but with similar clinical characteristics, Bess et al. proposed another mechanism by which “the magnitude of inner ear fluid vibrations that should stimulate the inner hair cells was decreased” (9). Thus, diminishing the inner ear fluid movement as a response to air conduction *stimuli* would only logically generate lower auditory thresholds and could therefore be interpreted as an inner ear conductive hearing loss since the bone conduction remains normal.

In the aftermath of the 1998 article by Minor et al. describing the first observations of SSC dehiscence (SSCD), the concealed mechanism of this type of inner ear conductive hearing loss was hypothesized (10). The term third window (TW) was originally used by Cawthorne in his personal description of SC fenestration in patients with otosclerosis and then adopted (4, 11–14); nowadays, it corresponds to a defect in the bony structure of the otic capsule, which locally reduces the local hydrodynamic resistance of the perilymphatic space (PS) (13).

Thereafter, Merchant and Rosowski theorized that an SSCD acts as a TW between the vestibule and the dura resulting in pseudo-conductive hearing loss through worsened airway thresholds and improved bone conduction (12). The labyrinthine membrane, unprotected by the bony covering at the level of the dehiscence, has therefore a locally higher compliance allowing a deviation of the perilymphatic flow toward the bony defect (10, 12, 15–18). Thus, under some conditions, depending on the location, size, and shape of the dehiscence, the normal acoustic energy transported by the PS could be transmitted to the vestibular compartment of the endolymphatic system (ES). On the one hand, according to the mechanism described above, if the TW is located in the anterior part of the labyrinthine PS, between the oval window and the round window, the consequences would be generally limited to a conductive/mixed hearing loss. On the other hand, if the TW is rather located toward the posterior part of the ES, then a vestibular co-stimulation by high-intensity sounds is possible, because the perilymphatic flow would no longer be directed entirely to the basilar membrane of the cochlea, but also partially to the vestibular compartment of the labyrinth (10, 12). As a result, a deformation of the membranous labyrinth will appear in the dehiscent area, modulated by sound characteristics (intensity, frequency). Then, due to a local transfer of vibrations by a resonance phenomenon between the perilymphatic and endolymphatic spaces, a significant deformation of the crista *ampullaris* can be observed (15, 16, 18). This anarchic vestibular co-stimulation mediated by strong sounds of varying intensities (15, 16, 18), e.g., the Tullio phenomenon, leads to vertigo or/and dizziness. This is one of the main clinical signs found in patients with SSCD as described by Minor in 1998 (10, 13, 15, 19).

### Recent Developments

Since the 2000s, we have witnessed the progressive appearance of TW variants with similar clinical and audiological features. The broader concept of “otic capsule dehiscence syndrome” (OCDS) proposed by Wackym et al. refers to all pathologies of the TW spectrum whose symptoms, clinical signs, and audiometric aspects correspond to bony defects of the otic capsule confirmed by high-resolution CT (HRCT) (20, 21). The same authors, however, reported a series of patients with a clinical presentation “specific” to an OCDS without corresponding radiodiagnosis evidence of dehiscence reminding to some degree of the concept of “mysterious deafnesses”' mentioned by Schuknecht (8, 21, 22).

Due to this progressive increase in new variants of OCD reported, the characterization of the anatomical structures involved, as well as the size and location of the TW on the otic capsule, has become essential for a better understanding of the various (or just slightly different) mechanisms associated with this pathology. This allows not only to systematize the different known variants and facilitate the diagnosis of “mysterious” conductive deafness (20–22), but also to propose new, potentially less invasive or more pathophysiological therapeutic strategies (23–26). The purpose of this article is: (1) to propose an anatomo-radiological classification of different OCD abnormalities and (2) to discuss the alleged pathomechanism of certain variants. Two case reports with multiple locations of OCD will also be discussed, including their clinical particularities, as well as the specific difficulties encountered in making a therapeutic decision.

## Materials and Methods

### Population

The clinical and radiological data of 259 patients diagnosed in our center with OCSD in the last 6 years were retrospectively reviewed. Patients with degenerative processes or chronic infection of the petrosal bone, whether they underwent surgery or not, were excluded.

### Vestibular and Audiological Evaluation

A standard neurotological examination, including cranial nerve evaluation and oto-microscopy, was routinely performed in all patients. Pure tone audiometry (PTA; Madsen Astera-Otometrics), middle ear reflexes (Madsen Zodiac 901 tympanometer), videonystagmography including bone vibratory test (BVT) and Valsalva maneuver (VNG, Ulmer System^®^ Synapsis SA), video head impulse test (VHIT, ICS Impulse^®^ GN Otometrics), cervical vestibular evoked potentials (cVEMPs) and ocular vestibular evoked potentials (oVEMPS) (Bio-Logic^®^ Nav-Pro system) in air conduction with 750 Hz stimuli were systematically performed in all patients.

### Radiological Evaluation

High-resolution CT (GE GSI Revolution, GE Healthcare, USA) of the petrous bone was performed in all patients. Slices were acquired helically in the axial plane at a nominal thickness of 0.625 mm with a 50% overlap of 0.312 mm, as recommended (27–29). Images were obtained in ultra-high resolution at 140 kV and 200 mAs/section. The primary images were reworked in the axial and coronal planes of the lateral CSC at a 60 mm field of view with a 512 matrix for an isometric voxel. Pöschl plane (i.e., superior SCC plane) using Advantage Workstation (AW) Server visualization software (GE Healthcare, USA) was also performed.

In addition, 3 Tesla MRI (3T MRI; GE Healthcare, Philips Ingenia, Philips healthcare) of the petrous bone and inner ear structures was also performed if associated pathologies were suspected or when vestibular and/or vascular structures appeared to be involved at the TW's interface. 3D T1-weighted contrast enhanced sequences allowed for confirmation of the vascular nature of the involved structure, and the HR 3D T2 labyrinth sequence DRIVE (DRIVen Equilibrium pulse, TE 157, TR 1000, slice thickness 0.4, Turbo factor 40, Matrix 500 × 500, voxel size: 0.4 × 0.4 isotropic) highlighted, when necessary, the morphology and permeability of the membranous labyrinth. Fused images between CT slices in Pöschl plane and 3D T1 weighted contrast enhanced sequence obtained with post-processing software (AW Server, GE Healthcare) were performed to assess the TW interface.

## Results

A total of 97 patients (40 men and 57 women) were included. One hundred and twenty ears with single or multiple (uni or bilateral) OCD locations were independently analyzed by a specialist radiologist and an expert otologist. Following this analysis, a classification of OCD was proposed based on the anatomic structures and radiological features involved at the TW partition (Table 1).

**Table 1:** Third Mobile Window Abnormalities (TMWA): classification and clinical elements.

	**Interface**	**Type**	**Number of patients**	**Clinical features**	**cVEMP thresholds**
Extralabyrinthine TMWA (OCD)	OC-Meningeal	I	48	Vertigo (42%) Auditory symptoms (35%)	Decreased (20%)
	OC-Vascular	II	28	Vertigo (64%) Auditory symptoms (64%)	Decreased (14%)
	OC-Petrosal	III	17	Vertigo (47%) Auditory symptoms (52%)	Decreased (21%)
Intralabyrinthine TMWA -like	Vestibular aqueduct - Posterior SC		4	Vertigo (50%) Auditory symptoms (25%)	Decreased (0%)
Multiple OCD	Multiple locations (on the same ear)	/	11	Vertigo (80%) Auditory symptoms (100%)	Decreased (40%)

*SC, Semicircular Canal; OCD, Otic capsule dehiscence*.

### Type I: OCD-Meningeal

**Figure 1:**
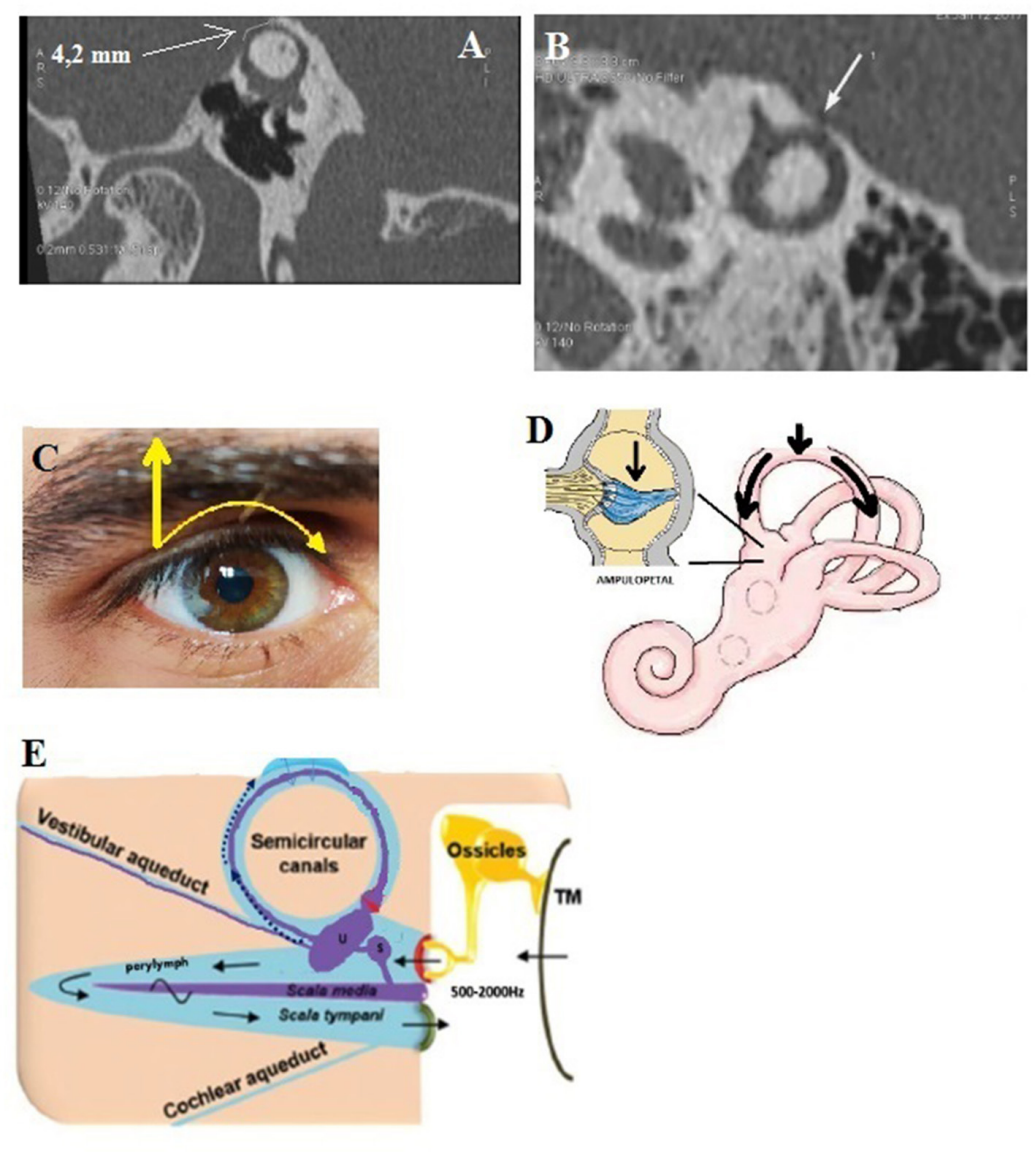
Type I extralabyrinthine otic capsule dehiscence (OCD) (OC-meningeal interface). **(A)** Superior semicircular canal dehiscence (SSCD); **(B)** PSCD. **(C,D)** Mechanical pressure (e.g., Valsalva against closed glottis) exerted at the dehiscence by the weight of the brain on the denuded membranous SSC inducing an inhibitory ampullopetal flow **(E)**. Tullio phenomenon: rapid nystagmic jerks in response to sounds at an intensity > 80 dB of frequency varying between 500 and 2,000 Hz.

This type (Figure 1) includes two main subsets that were the first cases of dehiscence described in the literature.

#### Type Ia

This type refers to the SSCD described by Minor, in which the SSC is typically in contact with the dura of the middle cerebral fossa (Figure 1A). Our series included 52 ears (24 right-sided and 28 left-sided) in 42 patients (21 M, 21 F) aged 2–85. Dehiscence was bilateral in 10 patients (4 M, 6 F).

#### Type Ib

This type of dehiscence involves the posterior SC (PSC) *which can be in contact or very close* to the dura of the posterior fossa (Figure 1B). It was found in 8 ears (5 right-sided, 3 left-sided) in 6 patients (4M, 2F), aged 48–64. Type Ib was present bilaterally in two patients (1M, 1F).

The pathophysiological mechanism of this type with its two sub-variants was largely described previously. In air conduction (sounds frequencies ranging from 500 to 2000 Hz) (15, 16, 18), the perilymph-driven hydraulic acoustic pressure, which normally reaches the round window, dissipates toward the dehiscence where a drop in impedance occurs, resulting in increased audiometric thresholds. According to Iversen and Rabbit, the resultant biomechanical phenomena in the membranous SC can lead to an opposite neural vestibular response at the level of the cupula (Figures 1C,D) depending on the frequency of the stimulus, with a decrease and increase of the afferent firing rate for low and high frequencies, respectively (15, 16). In bone conduction, the decrease in impedance favors the gradient between the vestibular and tympanic ramps and leads to a lowering of the thresholds. Application of a loud sound (Tullio phenomenon) (Figures 1D,E) or pressure in the external auditory canal (EAC) (Hennebert sign) potentially gives rise to an excitatory ampullofugal flow in the SSC. In addition, performing a Valsalva maneuver by pinching the nostrils classically results in ampullofugal movement (28). Ampullopetal (inhibitory) flow is then attained by applying negative pressure in the EAC, or from a closed glottis Valsalva maneuver (increased intracranial pressure) (Figure 1D).

### Type II: OCD-Vascular

**Figure 2:**
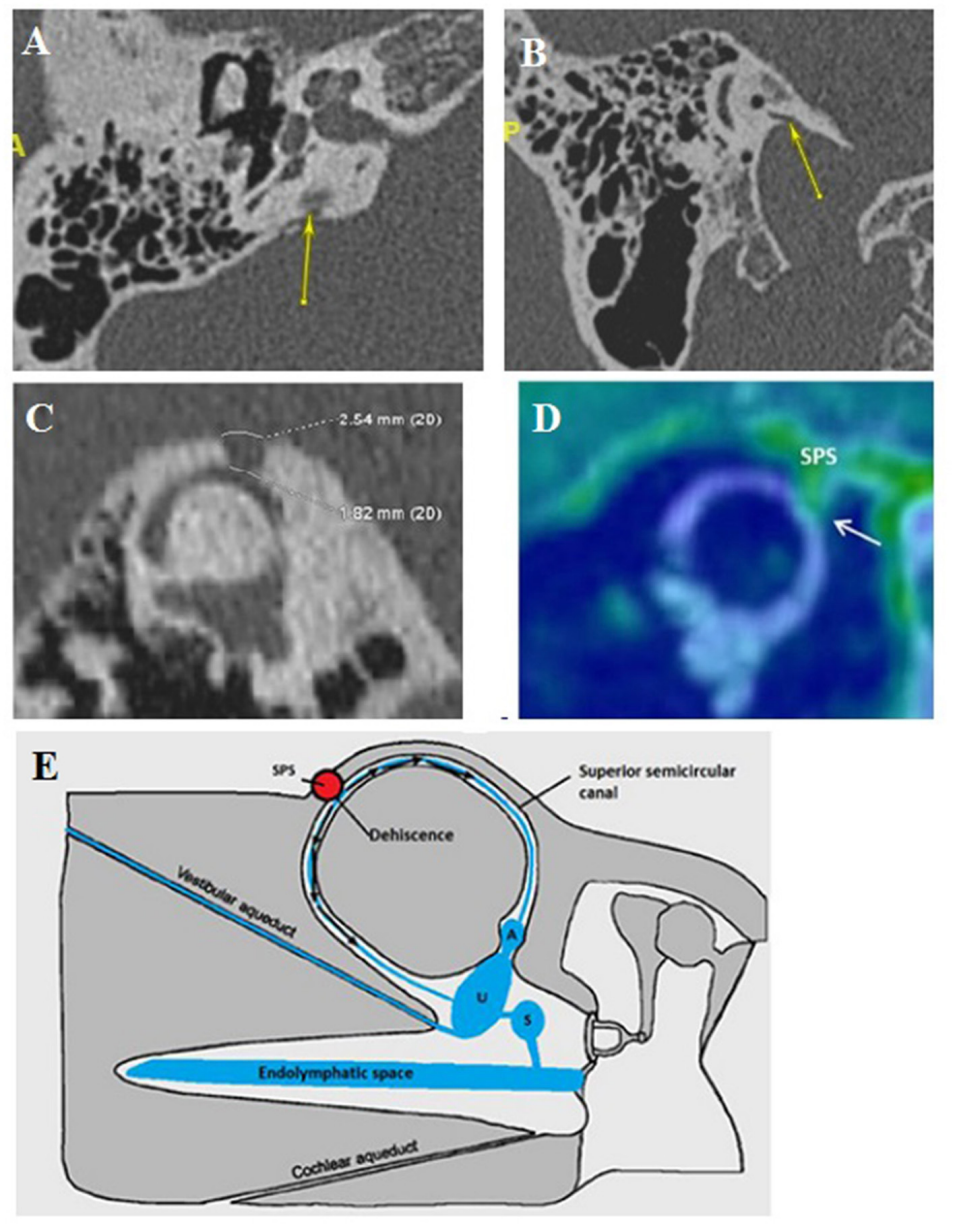
Type II extralabyrinthine OCD (OC-vascular interface). **(A,B)** High-resolution CT (HRCT) in axial plane (right), coronal plane (left): contact between the denuded VA and the IJV (yellow arrow). **(C,D)** Another patient: Right: HRCT in the plane of the superior (Poschl) denuded SC, and Left: 3T MRI labyrinthine, fused image between 3DT1-weighted contrast enhanced sequence and 3DT2 DRIVE sequence; mass effect exerted by the SPS against the membranous SC. **(E)** Proposed schematization of the mechanism of vestibulo-vascular TW. Pulsations of the interested vascular wall in intimate contact with the otic capsule membrane would cause non-physiological stimulation of the cochlea and/or the nearest vestibular sensory organs.

This type (Figure 2) of dehiscence correlates with a contact between the membranous vestibular or cochlear labyrinth and a vascular venous or, less frequently, arterial structure. It includes subclasses IIa, IIb, and IIc.

#### Type IIa

This type involves vasculo-vestibular contact between the membranous SSC and the superior petrous sinus (SPS). It was found in 8 ears (4 OD, 4 OG) in 6 patients (3M, 3F), aged 32–82 (Figures 2C,D). This presentation was found bilaterally in 2 patients (2F). Interestingly, there was no evidence of a “true” Tullio phenomenon including nystagmus elicited by loud sound stimulation in this group of patients. Moreover, the Valsalva maneuver against the closed glottis did not cause true vertigo except *slight* “dizziness” in a few cases. Instead, during this maneuver, an increase in the intensity of their pulsatile tinnitus was constantly reported. *This subtype can also integrate SSCD-subarcuate artery dehiscence, SSCD-superior petrosal vein dehiscence variants* (20).

#### Type IIb

Concern OCD involving the internal jugular vein (IJV) and various vestibular structures.

A dehiscence involving the vestibular aqueductus (VA) in contact with the IJV (Figures 2A,B) was found in 20 ears (14 right-sided, 6 left-sided), in 19 patients (6M, 13F), aged 11–72. The presentation was bilateral in one patient (1F). A dehiscence between IJV and PSC was found in 5 temporal bones (3 right-sided, 2 left-sided) in 4 patients (4F) aged 12–50. A dehiscence concerning the IJV and the cochlear *aqueductus* (CA) was found in 3 ears (left-sided) in 2 patients, age varying from 12 to 53 (1M, 1F).

In this subtype, the second most prevalent in our series as it was diagnosed in 19 from 97 patients, vertigo and/or pulsatile tinnitus induced by exertion were also constantly reported. Positional vertigo was also a commonly reported symptom with no evidence for a true benign positional paroxysmal vertigo (BPPV).

#### Type IIc

A contact between the membranous cochlea and the intrapetrous carotid artery was encountered in 1 ear (left-sided) in a 63-year-old female patient. Her main complaint was pulsatile tinnitus exerted by physical exercise synchronous with the peripheral pulse.

In this subtype, the pathomechanism of the inner ear structures stimulation does not seem obvious. However, it can be hypothesized that, compared to type I dehiscences, non-physiological audio-vestibular stimulation *can be* produced by the vascular structure (30) (Figure 2E). Thus, the vibrations generated by the vascular wall, in contact with the PS, will generate symptoms of intensity (pulsating tinnitus and/or dizziness) depending on the location, surface, and importance of any mass effect exerted by the vessel on the labyrinthine structure at TW (31).

### Type III: OCD-Petrosal *Bone*

#### Subtype IIIa

**Figure 3:**
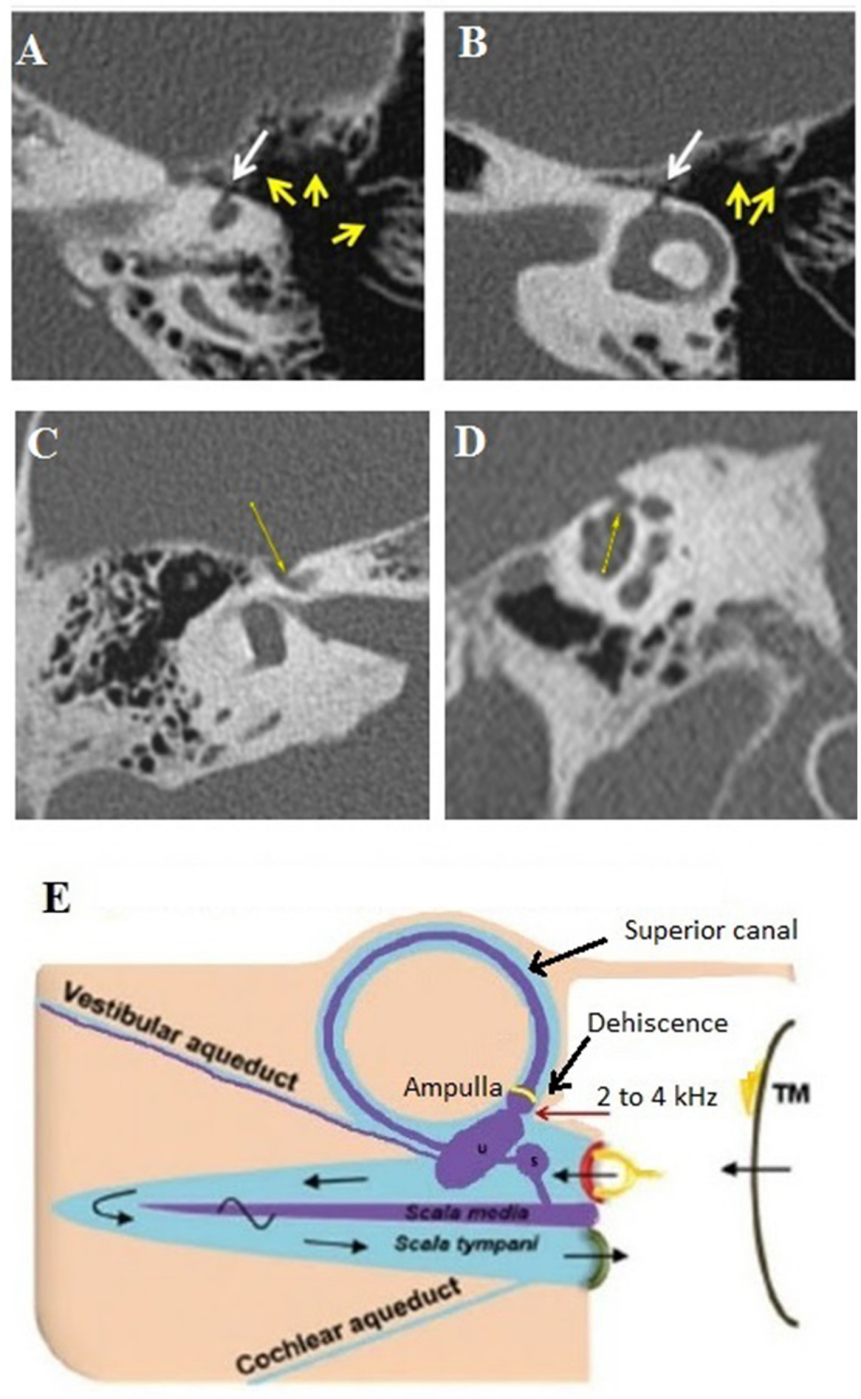
Type III extralabyrinthine OCD (OC-petrosal interface). **(A,B)** Ampullary dehiscence (white arrow) between the ampullary end of the LSC and the cells around otic capsule (yellow arrows). Note the hyperpneumatization of the mastoid and that the dehiscence limit extents to the LSC ampullae. **(C,D)** Right ear cochleo-facial dehiscence (CFD): the second turn of the cochlea dehiscent on the facial nerve canal in its geniculate zone on axial section **(C)** or coronal oblique section **(D)**. **(E)** Schema of the ampullary dehiscence (similar to a Helmholtz resonator) toward the tympanic cavity with stimulation of the cup by sound waves (red arrow). T M, tympanic membrane. Modified with permission from Merchant and Rosowski (12) and from Ho (13).

It involves a communication between the cochlea and the facial nerve canal or cochlear-facial dehiscence (CFD) (Figures 3C,D). This subtype was found in 18 ears (7 right-sided, 11 left-sided) in 15 patients (5M, 10F), aged 12–62. The presentation was bilateral in 3 cases (2M, 1F). In these patients, autophony and slight conductive hearing loss were predominant. Dizziness related to loud sounds or physical exercise was also described (see Table 1).

#### Subtype IIIb

It includes a dehiscent surface between the membranous labyrinthine and some hyperpneumatized mastoid air cells communicating with the tympanic cavity. It was encountered in one patient (a 60-year-old male, left side). A strong Tullio phenomenon associated with a typical down-beating nystagmus indicating a stimulation of the left SSC was highlighted by a left auditory stimulation at 120 dB between 2 and 4 kHz, although there was no conductive hearing loss.

Hyperpneumatization of the petrous bone appears to play an important role in the pathomechanism of this rare OCD. HRCT showed a significant number of large mastoid air cells communicating with the tympanic cavity (Figures 3A,B) and appears to be in intimate contact with the membranous SSC and the lateral SC (LSC), respectively, *via* an ampullary located dehiscence of maximum 1.5 mm width. The particular disposition of these mastoid air cells would act as an acoustic amplifier similar to the physical principle of a Helmholtz resonator (Figure 3E). Thus, the sound vibrations transmitted *via* the tympanic cavity and amplified at the mastoid cell/ampullary vestibular membrane interface will directly stimulate the cupula of the concerned SSC. As this hypothesis does not imply a significant acoustic energetic shunt toward the posterior labyrinth, therefore, it could explain the absence of conductive hearing loss. Although the lateral SC ampulla also appeared dehiscent (Figures 3A,B), most likely the air cells adjacent to this structure did not communicate with the tympanic cavity and, therefore, this SC remained asymptomatic.

#### Subtype IIIc

It includes cochlear (or labyrinthine) dehiscence over the internal auditory canal (IAC), a “near” dehiscence of this subtype is indicated in Figure 5C.

### Intralabyrinthine Third Mobile Window-Like Variants

**Figure 4:**
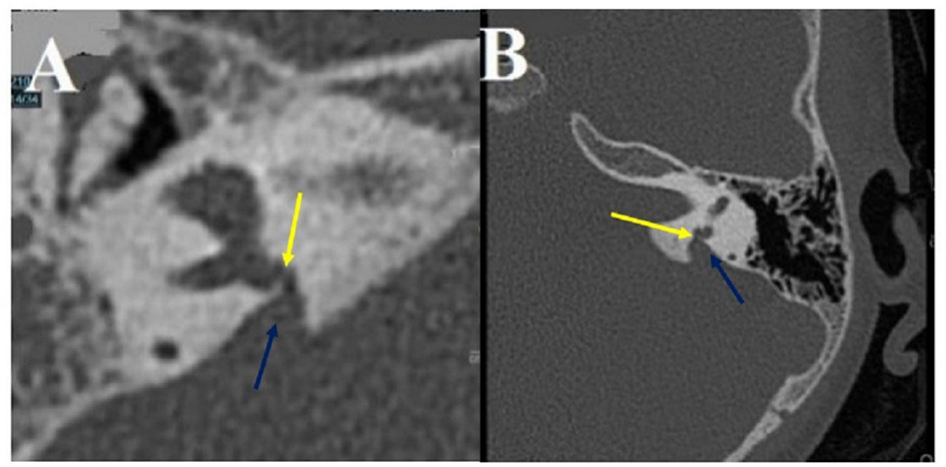
Intralabyrinthine TMWA-like. **(A,B)** Vestibulo-vestibular dehiscence: between the vestibular aqueduct widened to 3 mm (blue arrow) and the right posterior SC (yellow arrow) at the level of the common crus **(B)**.

This subgroup (Figures 4A,B) corresponds to an abnormal contact between two membranous parts of the same labyrinth being constantly associated with limited inner ear anomalies.

A dehiscence involving a dilated endolymphatic sac, in contact with the ampulla of the PSC, was found in 4 ears (2 right-sided, 2 left-sided) in 3 patients (3F), aged 3−30. Another similar case was noted involving an EVA in contact with the ampulla of the PSC. It is found in a single case of a left-sided ear in a 5-year-old boy.

As discussed below, some anatomical variants or other forms of intralabyrinthine TMWA sharing similar symptoms could be included in this subtype. Pathophysiological mechanisms including changes in endolymphatic flow caused by the presence of dilatation of the vestibular organs or the presence of intralabyrinthine obstacles (fibrosis, tumors) could be proposed.

### Case Report 1

**Figure 5:**
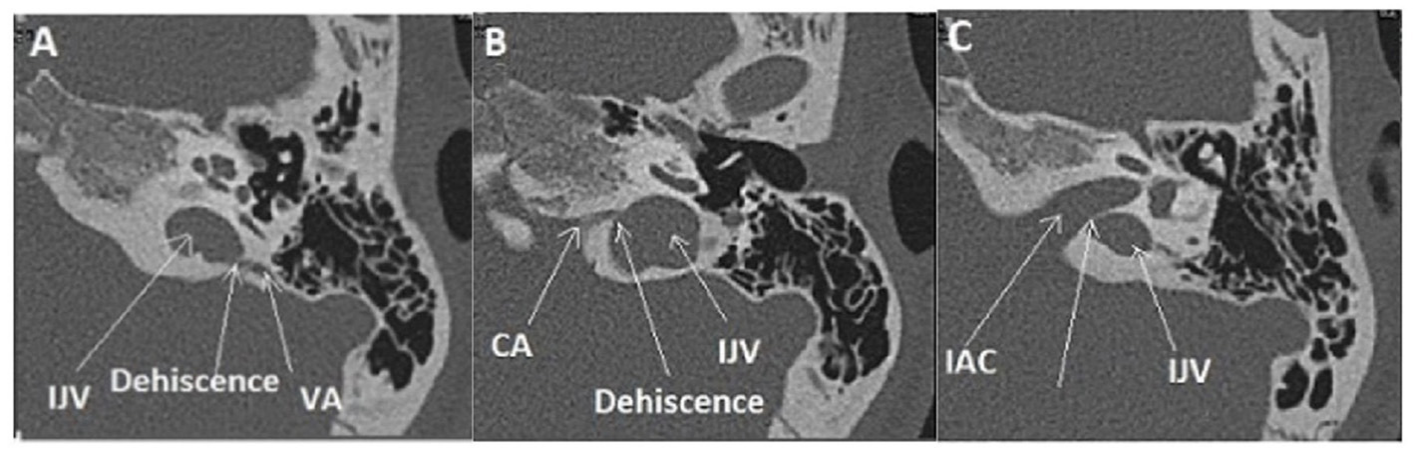
Multiple localization OCD (case report 1): high-riding left IJV at the origin of two type II of OCD. **(A)** Procidence of the IJV in the IAC (White arrow), a thin bone lamina is still remaining, **(B)** Dehiscence between IJV interface and VA. **(C)** Dehiscence between IJV interface and CA. IJV, internal jugular vein; IAC, internal auditory canal; VA, vestibular aqueduct; CA, cochlear aqueduct.

A 12-year-old child had a 5 years history of vertigo lasting for a few minutes associated with transient pulsatile tinnitus induced by physical effort. The oto-neurological examination and the audio-vestibular tests, including PTA, cVEMPs, VNG, and VHIT, were normal. The requested MRI for the IAC and inner ear structures did not reveal any pathological findings; however, HRCT showed a double OCD. While the first one was located between the left VA and the IJV (Figure 5A), the second has a slightly anterior localization, between the left CA and the same IJV (Figure 5B). Moreover, there was also a very thin bony lamina between the high-riding IJV and the IAC on the left side (*or a “near” subtype IIIc OCD*) white arrow (Figure 5C). This exceptional pediatric presentation needs close clinical follow-up, taking into account the limitation for moderate physical exertion experienced by this young patient.

### Case Report 2

**Figure 6:**
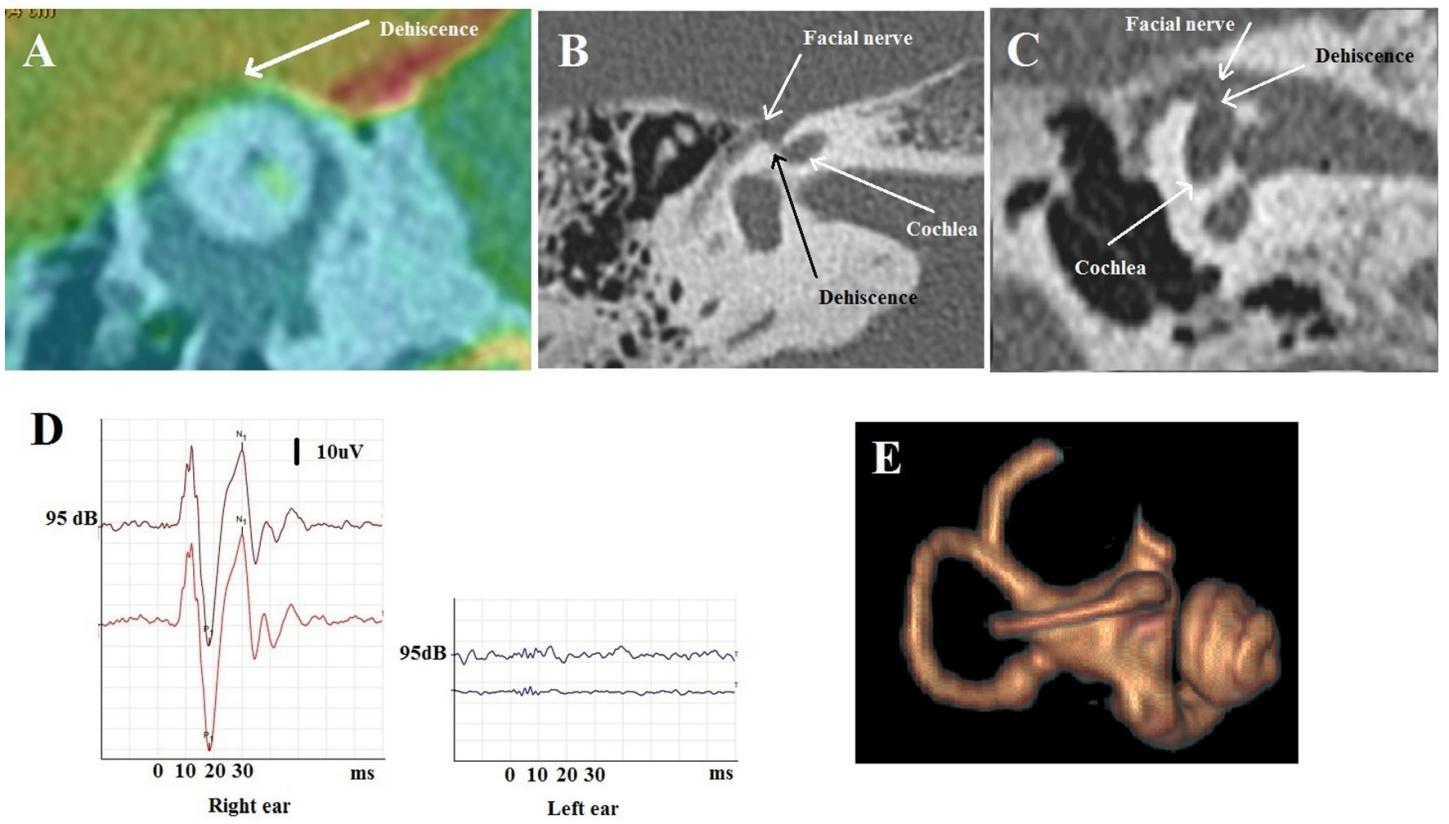
Multiple localization OCD (case report 2). **(A)** Fusion between HRCT and 3D T1 weighted contrast enhanced sequence in Poschl plane: right SSCD. **(B,C**) Temporal HRCT in axial **(B)** and coronal **(C)** plane: right CFD. **(D)** oVEMPs before surgery with large amplitude on the right side. **(E)** After surgery: labyrinthine MRI: partial amputation of the anterior arm and the top of the right SSC.

A 72-year-old patient suffered from vertigo, and induced by coughing pulsatile tinnitus. He was complaining of hearing the movements of his own eyelids blinking in the right ear. The pure tone audiometry showed a right-sided air-bone gap; cVEMPs revealed decreased threshold to 70 dB and oVEMPs revealed high amplitude potentials (100 uV) (Figure 6D). HRCT showed a right SSCD (type I OCD) (Figure 6A). As the patient was disabled by his symptoms, a surgical plugging of the SCC was proposed. Post-operatively, the patient complained of significant autophony and worsening of the pulsating tinnitus. PTA confirmed a deterioration of the audiometric thresholds in both bone and air conduction although ipsilateral oVEMPs disappeared and the cVEMPs threshold normalized. A labyrinthine MRI confirmed satisfactory plugging of the SCC (Figure 6E). While reexamining HRCT images, an ipsilateral CFD (type IIIa OCD) associated with the classical SSCD was revealed (Figures 6B,C). It was initially misdiagnosed by either the radiologist, the ENT surgeon, or by the neurotological staff as the SSCD was easily identified as “typical” and thus considered responsible for all symptoms.

In agreement with the patient, who refused any additional treatment, a simple audiological follow-up was proposed.

## Discussions

Although some systematization of TW have been proposed previously (11–14), we believe that proposing a new classification that takes into account not only the anatomical structures involved in the TW interface, but also their precise topographic location would lead to a better further understanding of the underlying pathophysiological mechanisms of this pathology. It is widely accepted that inframilimetric temporal bone HRCT with coronal or axial reconstructions is the “gold standard” to confirm the presence of an OCD. To date, labyrinthine MRI is generally used for post-operative follow-up to verify the effectiveness of the treatment, allowing signal interpretation of the residual fluid in the membranous SSC following canal plugging (32, 33). However, using 3D high-resolution MRI, a better exploration of anatomical structures involved at the site of the dehiscence is possible. In our experience, systematic combination of HRCT with 3T MRI would allow researchers to better understand some variants of TW's pathomechanisms, which appear as slightly different from that of the “well-known” SSCD. The implementation of this classification could also bring a potential addition to develop other treatment methods in the future. As an example, in case of a type II (vestibular-vascular) OCD showing a mass effect on the membranous labyrinth highlighted by 3D MRI, performing an endovascular procedure rather than performing a plugging-type surgery was simplest and with no expected vestibular or auditory impairments (24–26).

So far, it appears to be difficult to propose a correlation between symptoms or signs and each type or subtype of OCD, since there is a wide spectrum of clinical presentations that cannot be necessarily systematized (13, 14, 20). Some topographic classifications for OCD have already been suggested (11–14), but the involved anatomical structures have not been systematically taken into account. However, these have drawn the attention of clinicians to the existence of possible multiple localizations of OCD, not just the type I (a or b) OCD that is commonly sought by radiologists.

### Extralabyrinthine TMWA: OCD-Meningeal (Type I)

Type I OCD has been reported as the most common form of dehiscence (8%), followed by the PSC (1.2%), and less commonly the LSC (0.4%) (34, 35). Because it was suggested that LSC dehiscence has been associated with chronic otitis media and cholesteatoma as well as canal wall down mastoidectomy (36), we have excluded in our study all patients with otitic lesions or those with suspicion of perilymphatic fistula (PLF) in *relation with this chronic pathology*. The pathophysiological mechanisms of fistulae have already been mentioned above and have been extensively described elsewhere (16). Most patients in our series included in this subtype presented similar symptoms and/or signs as listed by Naert et al. for the SSCD (36).

### Extralabyrinthine TMWA: OCD-Vascular (Type II)

In this type of OCD, low frequency vascular wall pulses close to the infrasounds appear to be transmitted through the TW to the cochlear and/or vestibular sensors more or less efficiently (37, 38). It is likely that the extent of this acoustic stimulation depends on the shape and size of the TW, the mass effect exerted on labyrinthine structures by the vascular structures or factors, which may strengthen the intracranial blood flow. Therefore, the intensity of the resulting symptoms (pulsatile tinnitus, exertion-induced vertigo) seems to be well-correlated with the factors listed above.

The patients with SSCD by SPS (type IIa) presenting invalidating pulsatile tinnitus and dizziness exacerbated by physical exercise have already been reported (31, 39, 40). As for the dehiscence's involving the IJV bulb and labyrinthic membranous structures (VA and the PSC), Thenint et al. had already proposed in a limited series stenting of the IJV to limit the transmission of vascular vibrations to the labyrinth with good results (24). We proposed a similar approach in one of our patients with SSCD by SPS; a significant improvement was observed after the endovascular procedure, which consisted in stenting the SPS (25). Recently, a similar, although slightly modified, approach was used by another otologic group, since the SPS was just occluded (26) with a very good result. We recently emphasized the importance of combining the standard HRCT with 3T MRI allowing for better visualization of the membranous SSC and an eventual mass-effect by the adjacent SPS at the dehiscence level (31).

Interestingly, with a 750 Hz auditory tone burst stimulus, in type IIb dehiscences involving a VA-IJV interface, we did not find, as we would have expected, even in patients with severe symptoms, a systematic decrease in the cVEMP threshold. A possible explanation would be that the stimulation parameters currently used in the literature (short stimuli, frequency between 400 and 800 Hz) (40) do not allow the diffusion of low frequency acoustic energy (750 Hz) to the VA, which has a very thin wall (41). Future research could use more adequate frequency stimuli to ensure better diffusion of perilymph waves to the presumed VA-IJV interface with lower compliance. Moreover, Hu et al. proposed to associate HRCT slices with reconstructions to study the relationship between these two structures (42). These authors proposed a three-stage classification: no contact, contact without VA obstruction, and VA obstruction. They used MRI of the IACs to evaluate the presence of a *possible secondary* endolymphatic hydrops. When the VA was not conceived, in conjunction with a high-riding internal jugular bulb, a secondary hydrops was evoked.

Otic capsule -vascular involving the cochlea and the carotid artery (Type IIc) was rarely described in the literature (43). Our series included one observation.

### Extralabyrinthine TMWA: OCD-Petrosal (Type III)

Following the first description by Blake et al. of cochleo-facial dehiscence (CFD, subtype IIIa), Wackym et al. published a cohort of 16 patients with this type of OCD studying outcomes after surgical repair (20, 44). Surgical management with round window reinforcement in these patients was associated with improved symptoms and outcome. However, our findings matched only partially the clinical features reported in this study.

As stated above, the pathophysiological mechanism of subtype IIIb OCD seems distinct from the “classic” pathomechanism of SSCD. In our opinion, sound waves emerging through the ampullary dehiscence would generate a local perilymphatic flow directly transmitted to the endolymphatic compartment, stimulating the crista ampullaris by a local Helmholtz-like resonance phenomenon. This hypothesis allows, to some extent, for a similar explanation of TP as with vertigo in patients after fenestration surgery or in case of idiopathic fistula of the LSC (45, 46).

### Intralabyrinthine TMWA-Like Mechanism

In this type, we can assume there is an association with a coexisting developmental defect of inner ear structures. Our series includes several cases of EVA (five cases), associated with a dilated endolymphatic sac in contact with the VA through a bony dehiscence of the VA wall. Matsuda et al. recently reported the case of a congenital dehiscence of the stapes footplate in a patient presenting a sudden right-sided hearing loss and severe vertigo that occurred immediately after nose-blowing (47). These last mentioned variants, associated with challenging clinical pictures, allow us to insist and emphasize the importance of careful and collaborative study of audio-vestibular exams and imagery for the sake of finding the diagnosis in certain “unexplained” symptoms.

Some authors considered an isolated EVA or enlarged cochlear aqueduct as a distinct TW since the perilymphatic normal flow transporting the acoustic energy to the cochlear end receptors is disrupted (12). We agree with this vision although these pathological conditions are not generated by a “true” OCD, but the intimate mechanism seems quite similar to that of a third mobile window. Therefore, we could include these cases in the class “intralabyrinthine TMW” or having a TMW-like mechanism, in addition to intracochlear schwannomas (ICS) that could induce modifications of the endolymphatic flow. Indeed, in a cohort of 19 patients with ICS, Fröhlich et al. measured the cVEMPS thresholds (48). On the affected side, the threshold was unexpectedly lowered in 21% of patients mimicking the presence of a TMW. The authors suggested that individualizing the management of these patients with a detailed functional evaluation of the labyrinth is paramount to propose treatment options and predict outcomes. As a physiological explanation, the authors mentioned changes in endolymphatic flow secondary to tumor obstruction in a similar manner to endolymphatic hydrops. It has already been shown that some cases of endolymphatic hydrops can mimic the TW syndrome with a similar clinical presentation (49–52). Besides, primary overpressure in the endolymphatic or perilymphatic spaces could explain a limited conductive hearing loss as previously reported (53–55). It is worth adding here that the notion of “inner ear conductive hearing loss,” considered lately as specific to TW lesions, was already used by Muchnik et al. to describe the air bone gap (ABG) observed in some patients with Menière disease (53).

Other TMWA-like pathologies may include Perilymphatic fistula (PLF). Although it may appear anatomically similar to type III extralabyrinthine OCD, clinical evidence indicates the involvement of other endolymphatic flows generating nystagmus with different characteristics (56). The explanation for this difference may lie in the fact that, in PLF, the vestibular membrane is compromised at this level while in type III, it remains intact. Some authors have reconciled PLF with OCD because of similar pathophysiological elements (20, 57). Hence, PLFs have not been considered in our classification as “true TW” because they actually involve an opening of the membranous labyrinth that allows the leakage of perilymph and/or endolymph with the obvious direct negative impact on the vestibulo-cochlear micromechanics.

Similarly, Shim et al. published a series of ears with “cavitating otosclerosis” reaching the anterior wall of the IAC, looking at a poor correction of the ABG in the aftermath of a “stapedotomy” (58). The authors suggested an OCD-type mechanism not corrected by the stapedotomy to explain the lack of improvement of the ABG.

### Not Identified OCD (or CT-TMWA)

As introduced by Shuknecht in the early age of deafness surgery (8), Wackym et al. reported patients with a group of symptoms suggestive of OCD, even if the imaging was negative (21, 22). According to these newly described variants of OCD, performing temporal bone HRCT with infra-milimetric slice thickness as recommended can be of great benefit in the diagnostic process in such symptomatic patients and in search of all possible types of OCD (28, 59).

### Multiple OCD Localizations

When one site of OCD (extralabyrinthine TMWA) is confirmed, ENT specialists and radiologists should be aware of the risk of not diagnosing multiple localization. In our series, 11 of 97 patients presented with confirmed multiple localization OCD on the symptomatic side. See Table 2 for the most common symptoms found in multiple localization as well as the most common associations on the same ear; the most important audiological and vestibular data are also displayed. Besides an accurate and complete diagnosis, the main challenge in multiple OCDs in the same ear is to select an appropriate therapeutic strategy for patients with disabling symptoms. It also involves establishing the order in which these dehiscences should be treated. As per our knowledge, there is no available data in the literature to council practitioners about the approach of multiple OCDs.

**Table 2:** Clinical characteristics of patients with multiple localization OCD (all OCD were ipsilateral).

**Age**	**Ear**	**1st OCD dehiscence**	**2nd OCD dehiscence**	**Symptoms**	**Audiometry findings**	**cVEMPs**	**oVEMPs**
16	RE	PSC-IJV	CFD	Tinnitus with head movement Noise-induced vertigo	Mild Hearing loss ABG = 5 (RE)	Bilateral threshold (x2)	Higher amplitude (RE)
37	RE	SSC-SPS	CFD	Pulsatile tinnitus (RE)	Normal	Higher amplitude (LE)	Absent
48	LE	SSC-Meningeal	CFD	Noise-induced autophonia pulsatile tinnitus (LE)	Bilateral low-frequency hearing loss ABG = 5 bilateral	Higher amplitude (LE) Threshold 60 dB (LE)	Higher amplitude (LE) Threshold 60 dB (LE)
73	RE	SSC-Meningeal	CFD	Decreased hearing Tinnitus Autophonia Cough-induced vertigo	ABG = 30 dB (RE)	Higher amplitude (RE) Threshold 60 dB (RE)	Higher amplitude (RE) Absent (LE)
68	LE	SSC-Meningeal	Cochlea-Carotid	Decreased hearing	Mixed HL ABG = 50 dB (RE) SNHL (LE)	NA	NA
59	LE	IJV-Vestibular aqueduct	CFD	Tinnitus (tapping) (LE) Instability and vertigo	ABG = 10 dB (RE) 20 dB (LE)	Normal	NA
67	LE	IJV-Vestibular aqueduct	IJV-IAC	Pulsatile tinnitus (RE)	Normal	Normal (RE) Absent (LE)	NA
46	LE	SSC-Meningeal	CFD	Bilateral HL Tinnitus (RE) Effort-induced vertigo	ABG = 20 dB (RE) Bilateral SNHL	Absent (RE) Decreased threshold 60 dB (LE)	Absent (RE) Decreased threshold 70 dB (LE)
72	RE	SSC-Meningeal	IJV-Vestibular aqueduct	Autophonia Pulsatile tinnitus Effort-induced vertigo	Bilateral SNHL	Threshold 50 dB (RE) Normal (LE)	NA
13	LE	IJV-Vestibular aqueduct	IJV-Cochlear aqueduct	Effort-induced vertigo	Normal	Normal	NA

Case report 1 illustrates a case of multiple type II dehiscence localization on the same side, involving the IJV and the VA and the CA. We do not find current surgical techniques that would be appropriate to treat this pediatric patient. As the connection between these anatomical conditions and symptomatology appears evident, in case of clinical aggravation a reinforcement could be proposed by stenting the IJV wall as described by Thénint et al. (24).

**Figure 7:**
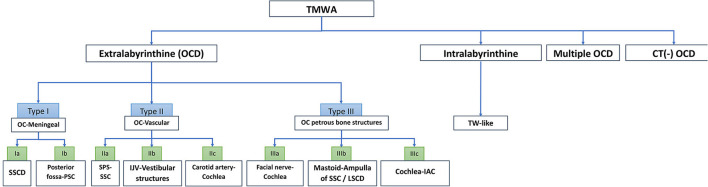
Algorithm of TMWA classification. IAC, internal auditory canal; IJV, internal jugular vein; LSCD, lateral semi-circular canal; OC, otic capsule; OCD, otic capsule dehiscence; SSCD, superior semi-circular canal dehiscence; SPS, superior petrosal sinus; PSC, posterior semi-circular canal; SSC, superior semi-circular canal; TW, third window; TMWA, third mobile window abnormalities.

In case report 2, HRCT showed a dehiscent right SSC in a patient with disabling symptoms. A surgical procedure by SSC plugging was proposed, which led to worsening of autophony, pulsatile tinnitus, and a significant aggravation of the initial conductive hearing loss. An ipsilateral CFD (type III), not initially detected, was secondarily discovered when the petrosal bone CT was reevaluated. This unexpected post-operatory aggravation might be due to a reinforcement of perilymphatic flow oriented toward the CFD level as a result of the membranous SSC plugging, which led to the exposure of the “fourth window,” clinically “silent” before surgery. In cases of CFD, reinforcement of the round window was suggested by Wackym et al. (20), although these authors do not mention it in case of multiple OCD localizations. In our opinion, plugging and RW reinforcement may be proposed in the same surgical intervention in deeply impaired patients. However, the results could be random, as the volumes and circulation of the inner ear fluids could be radically affected by surgery with unpredictable results on the initial symptoms. Given the apparently high risk of skipping a multiple localization, our group found it important to include it as “multiple OCD” (Table 1; Figure 7) in a separate branch of this classification.

### Perspectives

Superior semicircular dehiscence has been the subject of numerous articles codifying its surgical management (60). Concomitantly, with a better understanding of the OCD pathophysiology, new therapeutic procedures have emerged to diminish operative risks. Creighton et al. described the case of a patient with a SSCD who benefited from an endoscopic “underwater” procedure in a balanced salt solution (61). This attempt aimed at limiting the risk of PLF by injecting fluid into the mastoid as a counter pressure method during the plugging procedure.

From our perspective, the major principle to be considered in the future for the treatment of TW lesions would be to find the most appropriate methods that aim at reducing the abnormal transmission of sound vibrations through the abnormal window to the vestibular and/or to cochlear end organs, without excluding any highly functional labyrinthine segment. A step forward would possibly be the manufacturing of a physical or a numerical semicircular model, which would allow for a better pathophysiological approach and management of these challenging pathologies. With the actual constraints and ethical considerations in clinical medical research, this method could be promising. It will allow researchers to obtain an “almost-real” simulation with hydrodynamic modification analysis possibly caused by the surgical procedure type “plugging.” It might be the ideal way to manage and possibly solve the actual pathophysiologic dilemmas as for multiple location dehiscence.

## Conclusions

Based on anatomo-radiologic data of the inner ear structures involved, a classification of TMWA is proposed herein. Its aim is to help to conventionally systematize the increasing number of currently known variants of this pathology. Moreover, this classification could allow ENT specialists, radiologists, and/or clinical radiologists to better understand some OCD variants, as well as to imagine possible innovative therapeutic approaches in the future. In type II OCD, involving vascular structures, MRI has greatly contributed to a better visualization of the anatomical elements in contact at the level of the TW, which has been an essential element for the current classification and for the development of newly endovascular treatment techniques.

## Data Availability Statement

The raw data supporting the conclusions of this article will be made available by the authors, without undue reservation.

## Ethics Statement

The studies involving human participants were reviewed and approved by CUMG de Lyon. Written informed consent to participate in this study was provided by the participants' legal guardian/next of kin.

## Author Contributions

EI, PR, and SI: concept and design. EI, HT-V, and PB: supervision. EI, PR, and AL-B: resource and materials. EI, SI, and AP: data collection and processing. PR, EI, PB, and AL-B: analysis and interpretation. EI and PR: literature search. PR, EI, SI, and PB: writing. PB and HT-V: critical reviews. All authors contributed to the article and approved the submitted version.

## Conflict of Interest

The authors declare that the research was conducted in the absence of any commercial or financial relationships that could be construed as a potential conflict of interest.

## Publisher's Note

All claims expressed in this article are solely those of the authors and do not necessarily represent those of their affiliated organizations, or those of the publisher, the editors and the reviewers. Any product that may be evaluated in this article, or claim that may be made by its manufacturer, is not guaranteed or endorsed by the publisher.

## References

[1] Jenkins GJ. Serial microscopic sections of the labyrinth and middle ear, showing ankylosis of the stapes; otosclerosis. Proc R Soc Med. (1914) 7:40–1. 10.1177/003591571400701222 19978099 PMC2002686

[2] Sourdille M. New technique in the surgical treatment of severe and progressive deafness from otosclerosis. Bull N Y Acad Med. (1937) 13:673–91. 10.1288/00005537-193712000-00002 19312040 PMC1966137

[3] Lempert J. Lempert fenestra nov-ovalis operation for the restoration of serviceable unaided hearing in patients with clinical otosclerosis: its present evolutionary status. Arch Otolaryngol. (1947) 46:478–511. 10.1001/archotol.1947.00690020490003 20268766

[4] Cawthorne T. Otosclerosis. J Laryngol Otol. (1955) 69:437–56. 10.1017/S0022215100050933 13242969

[5] Holmgren G. The surgery of otosclerosis. Ann Otol Rhinol Laryngol. (1992) 101:546–55. 10.1177/000348949210100702 1626900

[6] Tullio P. Das Ohr und die Entstehung der Sprache und Schrift. Berlin: Urban and Schwarzenberg (1929).

[7] Addams-Williams J Wu K Ray J. The experiments behind the Tullio phenomenon. J Laryngol Otol. (2014) 128:223–7. 10.1017/S0022215114000280 24548750

[8] Schuknecht HF. Otologic mystery. Am J Otol. (1987) 8:182–3. 3591924

[9] Bess FH Miller GW Glasscock ME 3rd Bratt GW. Unexplained conductive hearing loss. South Med J. (1980) 73:335–8. 10.1097/00007611-198003000-00018 7361138

[10] Minor LB Solomon D Zinreich JS Zee DS. Sound- and/or pressure-induced vertigo due to bone dehiscence of the superior semicircular canal. Arch Otolaryngol Head Neck Surg. (1998) 124:249–58. 10.1001/archotol.124.3.249 9525507

[11] Minor LB Cremer PD Carey JP Della Santina CC Streubel SO Weg N. Symptoms and signs in superior canal dehiscence syndrome. Ann N Y Acad Sci. (2001) 942:259–73. 10.1111/j.1749-6632.2001.tb03751.x 11710468

[12] Merchant SN Rosowski JJ. Conductive hearing loss caused by third-window lesions of the inner ear. Otol Neurotol. (2008) 29:282–9. 10.1097/MAO.0b013e318161ab24 18223508 PMC2577191

[13] Ho ML. Third window lesions. Neuroimaging Clin N Am. (2019) 29:57–92. 10.1016/j.nic.2018.09.005 30466645

[14] Ho ML Moonis G Halpin CF Curtin HD. Spectrum of third window abnormalities: semicircular canal dehiscence and beyond. Am J Neuroradiol. (2017) 38:2–9. 10.3174/ajnr.A4922 27561833 PMC7963676

[15] Iversen MM Rabbitt RD. Wave mechanics of the vestibular semicircular canals. Biophys J. (2017) 113:1133–49. 10.1016/j.bpj.2017.08.001 28877495 PMC5658742

[16] Iversen MM Rabbitt RD. Biomechanisms of third window syndrome. Front Neurol. (2020) 11:891. 10.3389/fneur.2020.00891 32982922 PMC7477384

[17] Carey J Hirvonen T Minor L. Acoustic responses of vestibular afferents in a model of superior canal dehiscence. Otol Neurotol. (2004) 25:345–52. 10.1097/00129492-200405000-00024 15129116

[18] Grieser BJ Kleiser L Obrist D. Identifying mechanisms behind the tullio phenomenon: a computational study based on first principles. J Assoc Res Otolaryngol. (2016) 17:103–18. 10.1007/s10162-016-0553-0 26883248 PMC4791416

[19] Misun V. The standing acoustic wave principle within the frequency analysis of acoustic signals in the cochlea. J Biomed Eng Med Devic. (2016) 1:3. 10.4172/2475-7586.1000116

[20] Wackym PA Balaban CD Zhang P Siker DA Hundal JS. Third window syndrome: surgical management of cochlea-facial nerve dehiscence. Front Neurol. (2019) 10:1281. 10.3389/fneur.2019.01281 31920911 PMC6923767

[21] Wackym PA Wood SJ Siker DA Carter DM. Otic capsule dehiscence syndrome: superior semicircular canal dehiscence syndrome with no radiographically visible dehiscence. Ear Nose Throat J. (2015) 94:E8–24. 10.1177/014556131509400802 26322461

[22] Wackym PA Agrawal Y Ikezono T Balaban CD. Editorial: third window syndrome. Front Neurol. (2021) 12:704095. 10.3389/fneur.2021.704095 34220698 PMC8250852

[23] Ionescu EC Al Tamami N Neagu A Ltaief-Boudrigua A Gallego S Hermann R . Superior semicircular canal ampullae dehiscence as part of the spectrum of the third window abnormalities: a case study. Front Neurol. (2017) 8:683. 10.3389/fneur.2017.00683 29312118 PMC5742101

[24] Thénint MA Barbier C Hitier M Patron V Saleme S Courthéoux P. Endovascular treatment of symptomatic vestibular aqueduct dehiscence as a result of jugular bulb abnormalities. J Vasc Interv Radiol. (2014) 25:1816–20. 10.1016/j.jvir.2014.07.013 25442142

[25] Ionescu EC Coudert A Reynard P Truy E Thai-Van H Ltaief-Boudrigua A . Stenting the superior petrosal sinus in a patient with symptomatic superior semicircular canal dehiscence. Front Neurol. (2018) 9:689. 10.3389/fneur.2018.00689 30177909 PMC6110153

[26] Aw GE Parker GD Halmagyi GM Saxby AJ. Pulsatile tinnitus in superior semicircular canal dehiscence cured by endovascular coil occlusion of the superior petrosal sinus. Otol Neurotol. (2021) 42:e629–30. 10.1097/MAO.0000000000003012 33394940

[27] Belden CJ Weg N Minor LB Zinreich SJ. CT evaluation of bone dehiscence of the superior semicircular canal as a cause of sound and/or pressure-induced vertigo. Radiol. (2003) 226:337–43. 10.1148/radiol.2262010897 12563123

[28] Ward BK Carey JP Minor LB. Superior canal dehiscence syndrome: lessons from the first 20 years. Front Neurol. (2017) 8:177. 10.3389/fneur.2017.00177 28503164 PMC5408023

[29] Ward BK van de Berg R van Rompaey V Bisdorff A Hullar TE Welgampola MS . Superior semicircular canal dehiscence syndrome: diagnostic criteria consensus document of the committee for the classification of vestibular disorders of the Bárány Society. J Vestib Res. (2021) 31:131–41. 10.3233/VES-200004 33522990 PMC9249274

[30] Liu Z Bi W Li J Li Q Dong C Zhao P . Superior semicircular canal dehiscence in relation to the superior petrosal sinus: a potential cause of pulsatile tinnitus. Clin Radiol. (2015) 70:943–7. 10.1016/j.crad.2015.04.017 26072165

[31] Ionescu E Reynard P Coudert A Roiban L Boudrigua AL Thai-Van H. Superior semicircular canal dehiscence by superior petrosal sinus: proposal for classification. J Int Adv Otol. (2021) 17:35–41. 10.5152/iao.2020.9384 33605219 PMC7901425

[32] Lee SY Bae YJ Kim M Song JJ Choi BY Koo JW. Changes in vestibulo-ocular reflex gain after surgical plugging of superior semicircular canal dehiscence. Front Neurol. (2020) 11:694. 10.3389/fneur.2020.00694 32849185 PMC7385253

[33] Seroussi J Hautefort C Gillibert A Kania R Guichard JP Vitaux H et al. Postoperative MR imaging features after superior semicircular canal plugging in minor syndrome. Diagn Interv Imaging. (2018) 99:679–87. 10.1016/j.diii.2018.08.008 30220585

[34] Stimmer H Hamann KF Zeiter S Naumann A Rummeny EJ. Semicircular canal dehiscence in HR multislice computed tomography: distribution, frequency, and clinical relevance. Eur Arch Otorhinolaryngol. (2012) 269:475–80. 10.1007/s00405-011-1688-6 21739095

[35] Paladin AM Phillips GS Raske ME Sie KC. Labyrinthine dehiscence in a child. Pediatr Radiol. (2008) 38:348–50. 10.1007/s00247-007-0696-6 18066541

[36] Naert L Van de Berg R Van de Heyning P Bisdorff A Sharon JD Ward BK . Aggregating the symptoms of superior semicircular canal dehiscence syndrome. Laryngoscope. (2018) 128:1932–8. 10.1002/lary.27062 29280497

[37] Beggs C Tavoni V Menegatti E Tessari M Giovardini L Ragazzi R. Spectral characteristics of the internal jugular vein and central venous pressure pulses: a proof of concept study. Veins Lymphatics. (2021) 10:10–16. 10.4081/vl.2021.9732

[38] Koo JW Hong SK Kim DK Kim JS. Superior semicircular canal dehiscence syndrome by the superior petrosal sinus. J Neurol Neurosurg Psychiatry. (2010) 81:465–67. 10.1136/jnnp.2008.155564 20176603

[39] Schneiders SMD Rainsbury JW Hensen EF Irving RM. Superior petrosal sinus causing superior canal dehiscence syndrome. J Laryngol Otol. (2017) 131:593–7. 10.1017/S0022215117001013 28502274

[40] Rosengren SM Govender S Colebatch JG. The relative effectiveness of different stimulus waveforms in evoking VEMPs: significance of stimulus energy and frequency. J Vestibul Res. (2009) 19:33–40. 10.3233/VES-2009-0345 19893195

[41] Sando I Ikeda M. The vestibular aqueduct in patients with Meniere's disease: a temporal bone histopathological investigation. Acta Oto-Laryngol. (1984) 97:558–70. 10.3109/00016488409132934 6331708

[42] Hu J Peng A Deng K Huang C Wang Q Pan X . Value of CT and three-dimensional reconstruction revealing specific radiological signs for screening causative high jugular bulb in patients with Meniere's disease. BMC Med Imaging. (2020) 20:103. 10.1186/s12880-020-00504-0 32867723 PMC7460768

[43] Lund AD Palacios SD. Carotid artery-cochlear dehiscence: a review. Laryngoscope. (2011) 121:2658–60. 10.1002/lary.22391 22109767

[44] Blake DM Tomovic S Vazquez A Lee HJ Jyung RW. Cochlear-facial dehiscence–a newly described entity. Laryngoscope. (2014) 124:283–9. 10.1002/lary.24223 23712934

[45] Zhang LC Sha Y Dai CF. Another etiology for vertigo due to idiopathic lateral semicircular canal bony defect. Auris Nasus Larynx. (2011) 38:402–5. 10.1016/j.anl.2010.11.003 21216120

[46] Lempert J. Physiology of hearing; what have we learned about it following fenestration surgery? AMA Arch Otolaryngol. (1952) 56:101–13. 10.1001/archotol.1952.00710020120001 14943329

[47] Matsuda H Tanzawa Y Sekine T Matsumura T Saito S Shindo S . Congenital Membranous stapes footplate producing episodic pressure-induced perilymphatic fistula symptoms. Front Neurol. (2020) 11:585747. 10.3389/fneur.2020.585747 33240208 PMC7683612

[48] Fröhlich L Curthoys IS Kösling S Obrist D Rahne T Plontke SK. Cervical and ocular vestibular-evoked myogenic potentials in patients with intracochlear schwannomas. Front Neurol. (2020) 11:549817. 10.3389/fneur.2020.549817 33192980 PMC7655125

[49] Young YH Wu CC Wu CH. Augmentation of vestibular evoked myogenic potentials: an indication for distended saccular hydrops. Laryngoscope. (2002) 112:509–12. 10.1097/00005537-200203000-00019 12148863

[50] Taylor RL Zagami AS Gibson WP Black DA Watson SR Halmagyi MG . Vestibular evoked myogenic potentials to sound and vibration: characteristics in vestibular migraine that enable separation from Menière's disease. Cephalalgia. (2012) 32:213–25. 10.1177/0333102411434166 22259049

[51] Wen MH Cheng PW Young YH. Augmentation of ocular vestibular-evoked myogenic potentials via bone-conducted vibration stimuli in ménière disease. Otolaryngol Neck Surg. (2012) 146:797–803. 10.1177/0194599811433982 22237297

[52] Manzari L Tedesco AR Burgess AM Curthoys IS. Ocular and cervical vestibular-evoked myogenic potentials to bone conducted vibration in Ménière's disease during quiescence vs during acute attacks. Clin Neurophysiol. (2010) 121:1092–101. 10.1016/j.clinph.2010.02.003 20202901

[53] Muchnik C Hildesheimer M Rubinstein M Arenberg IK. Low frequency air-bone gap in Menière's disease without middle ear pathology. A preliminary report. Am J Otol. (1989) 10:1–4. 2719083

[54] Yetişer S Kertmen M. Cochlear conductive hearing loss in patients with Meniere's disease. Kulak Burun Bogaz Ihtis Derg. (2007) 17:18–21. 17483606

[55] Sugimoto S Yoshida T Teranishi M Okazaki Y Naganawa S Sone M. The relationship between endolymphatic hydrops in the vestibule and low-frequency air-bone gaps. Laryngoscope. (2018) 128:1658–62. 10.1002/lary.26898 29105767

[56] Helmchen C Gehrking E Gottschalk S . Persistence of perilymph fistula mechanism in a completely paretic posterior semicircular canal. J Neurol Neurosurg Psychiatr. (2005) 76:280–2. 10.1136/jnnp.2004.038083 15654053 PMC1739499

[57] Weinreich WM Carey JP. Perilymphatic fistulas and superior semi-circular canal dehiscence syndrome. Adv Otorhinolaryngol. (2019) 82:93–100. 10.1159/000490276 30947173

[58] Shim YJ Bae YJ An GS Lee K Kim Y Lee SY . Involvement of the internal auditory canal in subjects with cochlear otosclerosis. Otol Neurotol. (2019) 40:e186–90 10.1097/MAO.0000000000002144 30741893

[59] Curtin HD. Imaging of conductive hearing loss with a normal tympanic membrane. AJR Am J Roentgenol. (2016) 206:49–56. 10.2214/AJR.15.15060 26491893

[60] Mau C Kamal N Badeti S Reddy R Ying YM Jyung RW . Superior semicircular canal dehiscence: diagnosis and management. J Clin Neurosci. (2018) 48:58–65. 10.1016/j.jocn.2017.11.019 29224712

[61] Creighton F Jr Barber SR Ward BK Sharon JD Carey JP. Underwater endoscopic repair of superior canal dehiscence. Otol Neurotol. (2020) 41:560. 10.1097/MAO.0000000000002277 32176150

